# Influence of dose reduction and iterative reconstruction on CT calcium scores: a multi-manufacturer dynamic phantom study

**DOI:** 10.1007/s10554-017-1061-y

**Published:** 2017-01-19

**Authors:** N. R. van der Werf, M. J. Willemink, T. P. Willems, M. J. W. Greuter, T. Leiner

**Affiliations:** 1 Department of Radiology, University Medical Center Groningen, University of Groningen, Hanzeplein 1, 9713 GZ Groningen, The Netherlands; 2 Department of Radiology, University Medical Center Utrecht, Heidelberglaan 100, 3584 CX Utrecht, The Netherlands

**Keywords:** Computed tomography, Coronary calcium, Calcium score, Low dose CT, Iterative reconstruction CT

## Abstract

To evaluate the influence of dose reduction in combination with iterative reconstruction (IR) on coronary calcium scores (CCS) in a dynamic phantom on state-of-the-art CT systems from different manufacturers. Calcified inserts in an anthropomorphic chest phantom were translated at 20 mm/s corresponding to heart rates between 60 and 75 bpm. The inserts were scanned five times with routinely used CCS protocols at reference dose and 40 and 80% dose reduction on four high-end CT systems. Filtered back projection (FBP) and increasing levels of IR were applied. Noise levels were determined. CCS, quantified as Agatston and mass scores, were compared to physical mass and scores at FBP reference dose. For the reference dose in combination with FBP, noise level variation between CT systems was less than 18%. Decreasing dose almost always resulted in increased CCS, while at increased levels of IR, CCS decreased again. The influence of IR on CCS was smaller than the influence of dose reduction. At reference dose, physical mass was underestimated 3–30%. All CT systems showed similar CCS at 40% dose reduction in combinations with specific reconstructions. For some CT systems CCS was not affected at 80% dose reduction, in combination with IR. This multivendor study showed that radiation dose reductions of 40% did not influence CCS in a dynamic phantom using state-of-the-art CT systems in combination with specific reconstruction settings. Dose reduction resulted in increased noise and consequently increased CCS, whereas increased IR resulted in decreased CCS.

## Introduction

The coronary calcium score (CCS) is known to be a strong predictor for major adverse cardiovascular events [1, 2]. Computed tomography (CT) is the first modality of choice for assessment of the presence and quantification of calcium in the coronary arteries. The number of CCS examinations with CT is expanding rapidly [3]. However, due to the expanding use of ionizing radiation in medicine, CT has become the main source of increased population dose in Western countries [4]. This dose issue is especially important when considering the 2013 guidelines from the American Heart Association that recommend CCS measurements if, after quantitative risk assessment, the risk-based treatment decision is uncertain in asymptomatic adults at intermediate and low-to-intermediate risk [5].

Recently, iterative reconstruction (IR) has become widely available on commercially available CT systems. IR allows for a dose reduction without the typical decrease in image quality [6–8]. It may therefore be possible to quantify CCS at lower dose levels, when using IR. Recent studies found that application of IR can result in spurious decreases in CCS in comparison with conventionally used filtered back projection (FBP) [9–11]. These effects of dose reduction and IR on CCS can be explained by their effect on image noise. At decreased dose an increase in noise is expected. This increase in noise can be associated with an increase in voxels above the calcium threshold of 130 Hounsfield Units (HU), which in turn increases CCS. Conversely, a decrease in CCS is expected with IR since it reduces noise [12–15].

Moreover, cardiac motion imposes problems for the stability of CCS since calcium can be blurred and CCS can be over- or underestimated, depending on the density of the calcification [16–18]. The combined effects of dose reduction, IR and heart rate on CCS for all major manufacturers have not been investigated before in a phantom study.

Therefore, the objective of this study was to evaluate the influence of dose reduction in combination with IR on CCS of moving calcifications in coronary CT on state-of-the-art CT systems from different manufacturers.

## Materials and methods

An anthropomorphic chest phantom (Thorax, QRM, Moehrendorf, Germany) with artificial lungs, a spine insert and a shell of soft tissue equivalent material was used [16, 17]. An extension ring of tissue equivalent material was placed around the chest to simulate an averaged sized patient of 400 × 300 mm (QRM-Extensionring, QRM, Germany) [19]. The center compartment of the phantom was filled with water in which a motion simulator (Sim2D, QRM, Moehrendorf, Germany) translated an artificial coronary artery with two calcium hydroxyapatite (HA) inserts. The inserts had densities of 196 ± 3, 380 ± 2, 408 ± 2 and 800 ± 2 mg HA/cm^3^ and masses of 38.5 ± 1.7, 74.6 ± 3.1, 80.1 ± 3.3 and 157.1 ± 6.5 mg HA, respectively (Appendices 2, 3).

All inserts had equal dimensions; length 10.0 ± 0.1 mm, diameter 5.0 ± 0.1 mm, volume 196.3 ± 8.1 mm^3^. The artificial arteries were linearly translated in the horizontal plane at a velocity of 20 mm/s perpendicular to the scan direction. This velocity is comparable to typical velocities of the left anterior descending and right coronary arteries during the late diastolic scan phase of the R-R interval, at heart rates between 60 and 75 bpm [20, 21].

In order to assess the influence of IR and dose reduction on CCS in a clinical setting, daily used clinical CT protocols for coronary calcium scoring were used. These protocols were equal to the vendor recommended protocols if available or were adapted based on recommendation by the specific manufacturer consultants. Four different state-of-the-art CT systems (referred to as S1–S4) were used: Discovery CT 750 HD (GE Healthcare, Waukesha, Wisconsin, USA), Brilliance iCT (Philips Healthcare, Best, The Netherlands), Somatom Definition Flash (Siemens Healthcare, Forchheim, Germany) and Aquilion One (Toshiba Medical Systems, Otawara, Japan), respectively (Table 1).

**Table 1:** Acquisition and reconstruction parameters used on CT system S1–S4

CT system	S1	S2	S3	S4
Tube voltage (kV)	120	120	120	120
Tube charge per rotation (mA)	500	185	285	230
Collimation (mm)	64 × 0.625	128 × 0.625	128 × 0.6	320 × 0.5
Rotation time (s)	0.35	0.27	0.28	0.35
Temporal resolution^a^ (ms)	175	135	75	175
Slice thickness (mm)	2.5	3.0	3.0	3.0
Increment (mm)	2.5	3.0	3.0	3.0
Kernel	Standard	XCA	B35f	FC12
Levels of IR	20, 60, 100%	1, 5, 7	1, 3, 5	weak, standard, strong
Noise level (HU)	26	22	28	24
CTDI_vol_ (mGy)	10.6	3.2	2.8	6.5
Software	Smartscore 4.0	Heartbeat-CS	Syngo	Vitrea FX 6.5.0

^a^As defined in the isocenter

The phantom was scanned at three dose levels by reduction of the tube current: a reference dose at 100% tube current, and at reduced dose levels of 40 and 80% reduced tube current. Each scan was repeated five times with a small translation (2 mm) and rotation (2°) between each scan by manually repositioning the phantom. The internal ECG signal of the motion controller was used to simulate the heart rate of the patient and used as ECG trigger on all four CT systems. The triggering was carefully timed so that data acquisition was during linear motion of the phantom.

Images were reconstructed with FBP, and three increasing levels of IR: the lowest (L1), an intermediate (L2) and the highest level available on the CT system (L3) (Table 1). For each data set the noise level in the images was assessed by calculating the standard deviation in the average Hounsfield value in a uniform water region. The amount of calcium of each insert was quantified as Agatston and mass scores with manufacturer-recommended software (Table 1) with a default threshold of 130 Hounsfield units (HU). A semi-automatic method was used for selecting the calcification by one observer. On each CT system, the mass score calibration factors were determined as described by McCollough et al. [19]. Although mass scores are not used clinically, they were included for this study because of its potential to compare the score to the physical mass.

The design of this study resulted in 480 calcium scores per CT system (5 acquisitions at 3 dose levels with 4 reconstruction types for 4 calcifications and 2 calcium scores).

Agatston score and mass score were expressed as median and 25th–75th percentile for each calcification insert and CT system. For each insert, CCS from both the iteratively reconstructed and FBP reconstructed data sets for reduced dose levels were compared to the CCS from the FBP reconstructed data sets at reference dose using a Wilcoxon signed rank test. All statistical analyses were performed with SPSS for Windows, version 22.0. A p value of 0.05 was used to determine significant differences.

## Results

### Influence of dose reduction and iterative reconstruction on noise

**Fig. 1:**
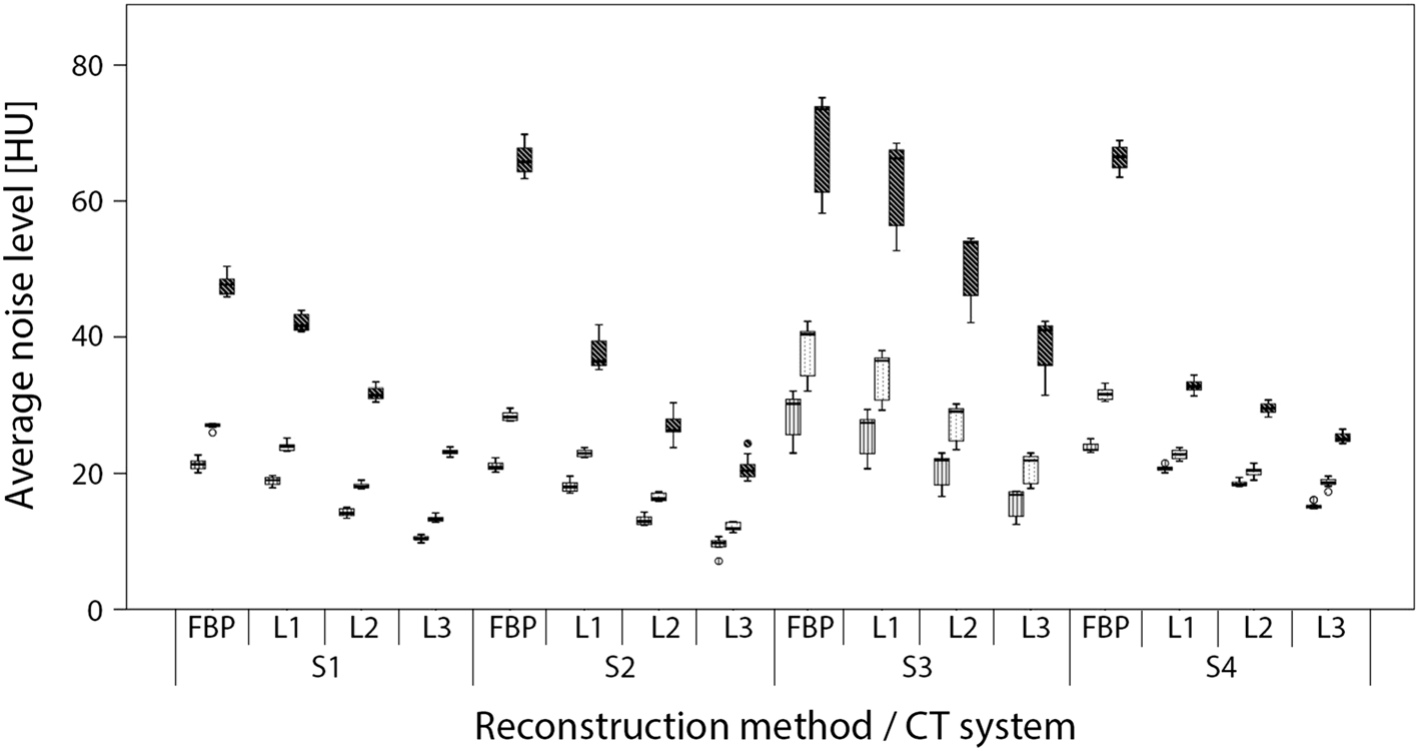
Average noise levels in a uniform water region for images reconstructed with filtered back projection (FBP) and increasing levels of IR L1, L2 and L3 as measured on CT systems S1, S2, S3 and S4. For each combination of reconstruction method and CT system box plots are shown for the average noise level at reference dose, and 40 and 80% reduced dose

For all CT systems and all reconstructions, a decrease in dose resulted in a vendor dependent increase in noise, whereas IR led to a decrease in noise (Fig. 1). Also, although the CTDI_vol_ differed at most with a factor of 3.8 between the CT systems, the noise levels varied less than 18% at FBP reference dose.

### Influence of dose reduction on Agatston score with FBP

**Fig. 2:**
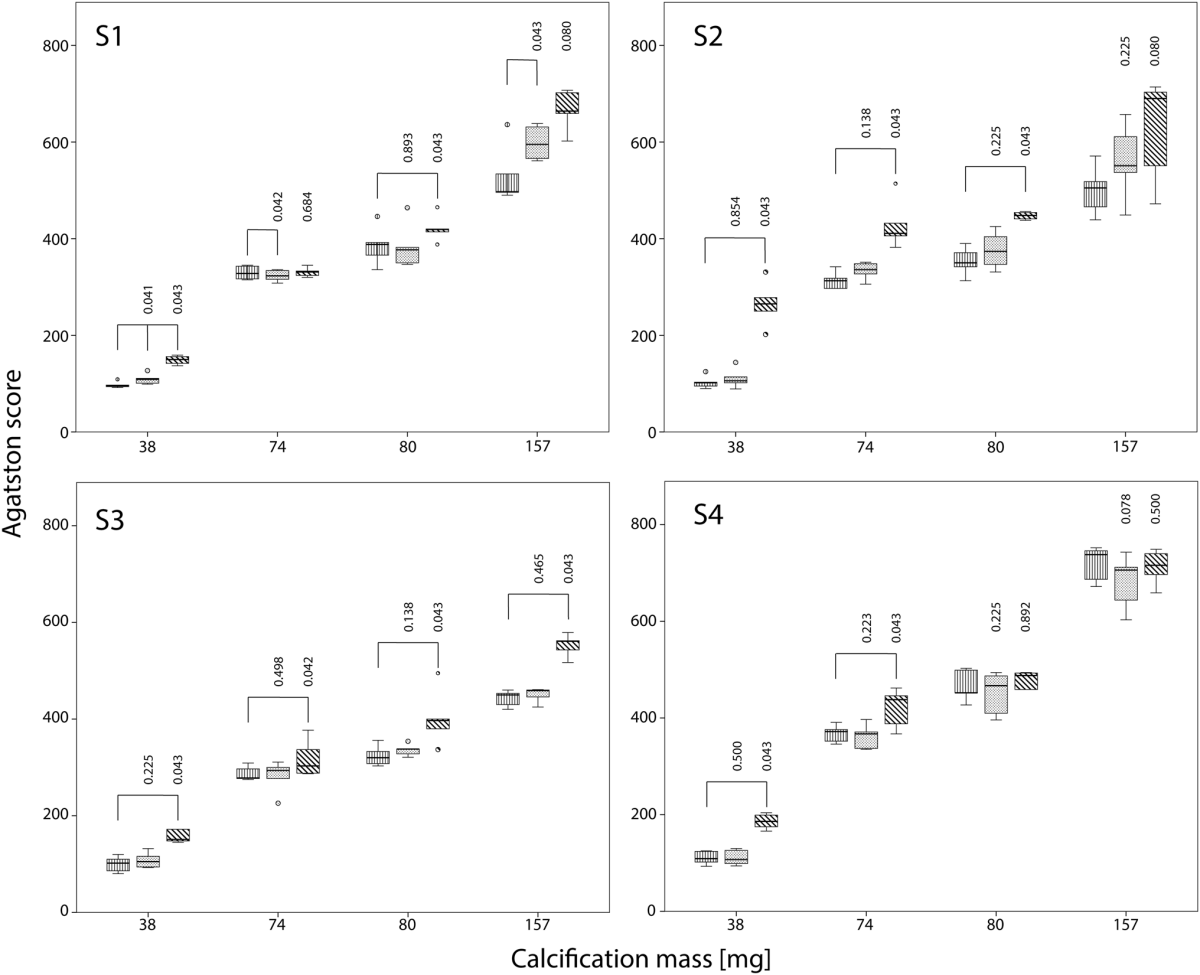
Influence of dose reduction on Agatston score for S1–S4 with FBP. The movement of the calcification corresponds to displacements seen with heart rates of 60–75 bpm. For calcifications of 38, 74, 80 and 157 mg box plots of the Agatston score at reference dose, and at 40 and 80% reduced dose are shown. Agatston scores are compared with the Agatston score at reference dose using the Wilcoxon signed rank test. Significant different Agatston scores are indicated by *brackets*

**Fig. 3:**
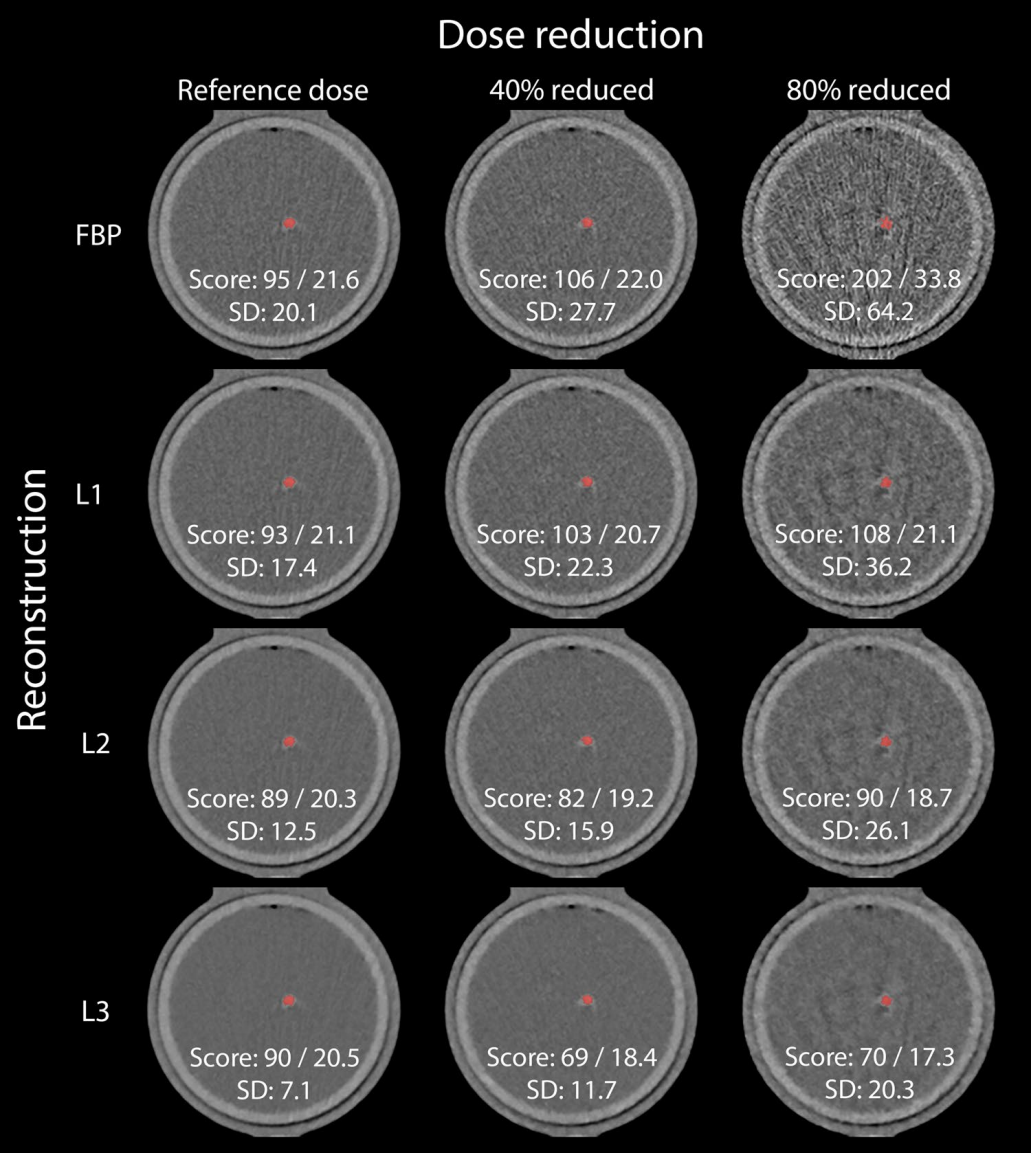
Reconstructed images of a 3.0 mm slice of the 38 mg insert moving at 20 mm/s on S2. Data was acquired at reference dose, and at 40 and 80% dose reduction (from *left* to *right*) and reconstructed with filtered back projection and increasing levels of IR L1, L2 and L3 (from *top* to *bottom*). CCS were included as Agatston score/mass score. Noise levels (SD) are expressed as Hounsfield Units. Window center was 90 HU and window width was 750 HU

Dose reduction resulted in significant increases in Agatston scores for almost all calcifications and CT systems (Fig. 2). This increase, in combination with an increase in noise, is depicted in the top row of Fig. 3.

For S1 at FBP and averaged over all inserts, Agatston scores increased by 8 and 25% at 40 and 80% reduced dose respectively. For the other CT systems similar increases in Agatston scores at FBP were observed at reduced dose with a corresponding average increase of 7 and 64% for S2, 4 and 26% for S3, and 1 and 23% for S4. The largest increase in Agatston score at reduced dose was observed for the 38 mg insert at 80% dose reduction: 58, 160, 48, and 71 for S1–S4, respectively.

### Influence of dose reduction on mass score with FBP

**Fig. 4:**
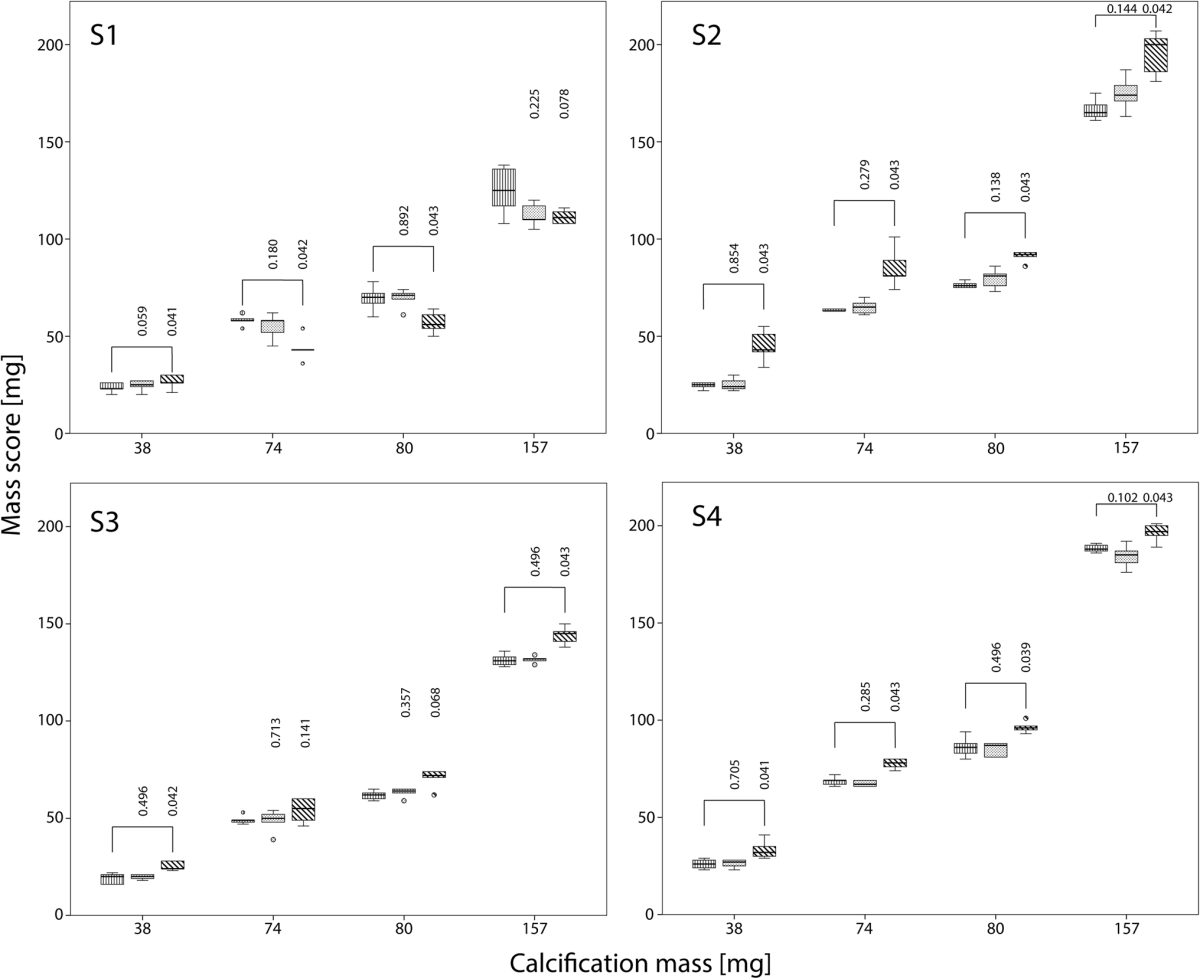
Influence of dose reduction on mass score for S1–S4 with FBP. The movement of the calcification corresponds to displacements seen with heart rates of 60–75 bpm. For calcifications of 38, 74, 80 and 157 mg boxplots of the mass score at reference dose, and at 40 and 80% reduced dose are shown. Mass scores are compared with the mass score at reference dose using the Wilcoxon signed rank test. Significant different mass scores are indicated by *brackets*

Also, dose reductions resulted in significantly increased mass scores at FBP for almost all inserts and CT systems, albeit that the increase was smaller than the increase in Agatston scores (Fig. 4).

At 40% reduced dose, mass scores increased on average by 0, 3, 1 and 0% for S1–S4 respectively in comparison with the mass score at reference dose. At 80% reduced dose, mass scores increased 35, 15 and 13% for S2–S4, whereas for S1 the mass score decreased 11%.

### Influence of iterative reconstruction on Agatston scores

**Fig. 5:**
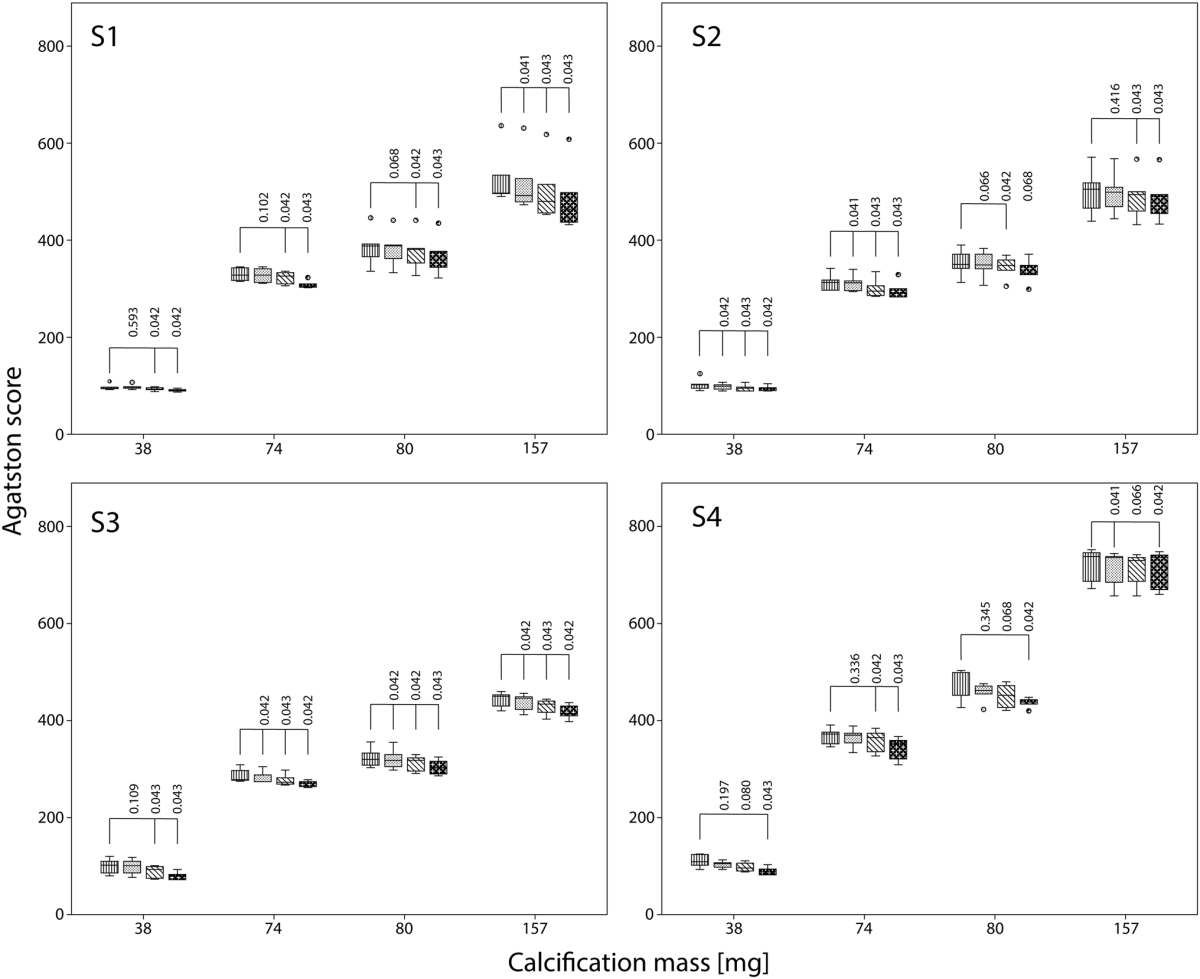
Influence of IR on Agatston score for S1–S4 with FBP. The movement of the calcification corresponds to displacements seen with heart rates of 60–75 bpm. For calcifications of 38, 74, 80 and 157 mg boxplots of the Agatston score at FBP and increasing levels of IR (from *left* to *right*: L1, L2 and L3) are shown. Agatston scores are compared with the Agatston score at FBP using the Wilcoxon signed rank test. Significant different Agatston scores are indicated by *brackets*

With increased IR levels, a significant decrease in Agatston scores was observed for almost all calcifications and CT systems (Fig. 5). This decrease in Agatston score was accompanied by decrease in noise, as can be seen from the left column in Fig. 3.

Averaged over all inserts, Agatston scores for S1 decreased on average 0, 2 and 5% at L1–L3 respectively. For S2 the corresponding decrease was 1, 4, and 5%; for S3 1, 4, and 9% and for S4 1, 4, and 7%. The largest decrease in Agatston score was again observed for the 38 mg calcification: 22% with L3 on S3, and 19% with L3 on S4.

### Influence of iterative reconstruction on mass scores

**Fig. 6:**
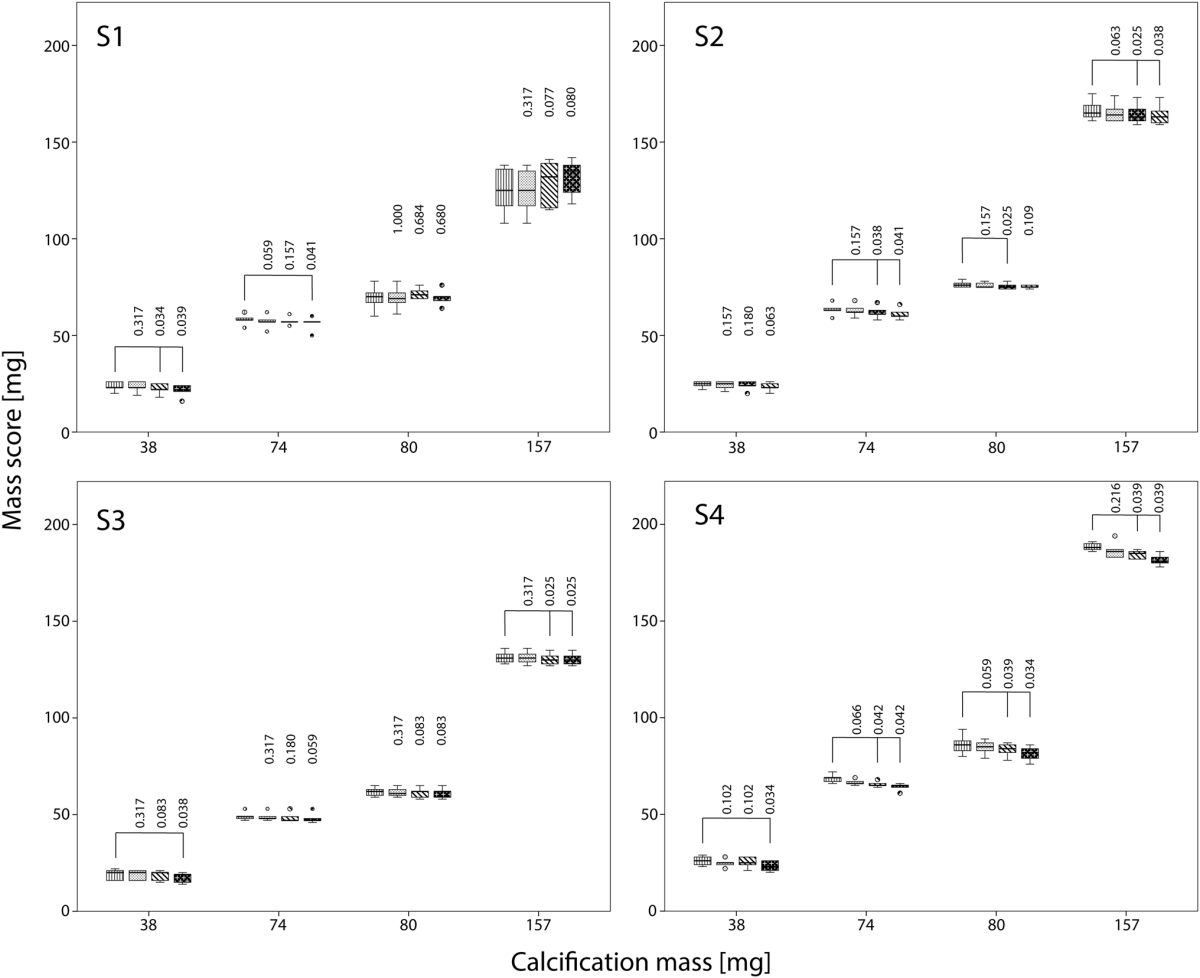
Influence of IR on mass score for S1–S4 with FBP. The movement of the calcification corresponds to displacements seen with heart rates of 60–75 bpm. For calcifications of 38, 74, 80 and 157 mg boxplots of the mass score at FBP and increasing levels of IR (from *left* to *right*: L1, L2 and L3) are shown. Mass scores are compared with the mass score at FBP using the Wilcoxon signed rank test. Significant different mass scores are indicated by *brackets*

The decrease in mass scores at increased levels of IR was smaller than the observed decrease in Agatston scores (Fig. 6). Mass score decreased on average between 0 and 6% for all CT systems and inserts.

### Combination of dose reduction and iterative reconstruction on Agatston and mass scores

Representative images of the reconstructed datasets are shown in Fig. 3.

For all four CT systems 40% dose reduction in combination with varying levels of IR did not result in significantly different Agatston and mass scores with respect to the reference dose (Table 2). For 80% dose reduction, only S2 in combination with L2 and L3 did not result in significantly different Agatston scores. For the other CT systems, there was no combination of investigated imaging parameters that resulted in Agatston scores which were unchanged from the reference protocol and dose.

**Table 2:** Reconstructions per CT system S1–S4 that did not result in significantly different Agatston and mass scores at 60–75 bpm and at a dose reduction of 40 and 80% with respect to the FBP-reference dose

CT system	Dose reduction (%)	Agatston score	Mass scores
S1	40	L1	FBP, L1
	80	n/a	n/a
S2	40	FBP, L1, L2, L3	FBP, L1, L2, L3
	80	L2, L3	L1
S3	40	FBP, L1, L2	FBP, L1, L2, L3
	80	n/a	L2, L3
S4	40	FBP	FBP
	80	n/a	n/a

*FBP*  filtered back projection, *L1, L2, L3* increasing levels of iterative reconstruction

On all CT systems, mass scores generally underestimated the physical mass of the calcifications. Mass scores at FBP and reference dose and deviations from the physical mass are listed in Table 3. Averaged over all inserts the physical mass was underestimated by 23, 12, 30, and 3% for S1–S4 respectively. The largest underestimation was again observed for the 38 mg insert, where the underestimation was 39, 33, 30, and 31%, respectively for S1–S4. At 40% reduced dose the underestimation was 24, 9, 29, and 3%. At 80% reduced dose the underestimation was 24 and 29% on S1 and S3, whereas S2 and S4 showed an overestimation of on average 16 and 9%. The influence of IR on mass scores was relatively small compared to the influence of dose reduction. At the maximum IR level, the underestimation of the physical mass at reference dose was 23, 15, 32, and 8% for S1–S4, respectively (averaged over all inserts).

**Table 3:** Physical mass and corresponding mass scores for all CT systems and calcification masses

CT system	Physical mass (mg)	Mass score (mg)	Deviation (%)
S1	38	23 (20–26)	−39 (−47; −32)
	74	58 (54–62)	−22 (−27; −16)
	80	70 (60–78)	−13 (−25; −3)
	157	125 (108–138)	−20 (−31; −12)
S2	38	25 (22–26)	−33 (−43; −31)
	74	63 (59–68)	−15 (−20; −8)
	80	76 (75–79)	−5 (−6; −1)
	157	165 (161–175)	5 (3; 11)
S3	38	20 (16–22)	−46 (−57; −42)
	74	49 (47–53)	−34 (−37; −28)
	80	62 (59–65)	−23 (−26; −19)
	157	131 (128–136)	−17 (−19; −13)
S4	38	26 (23–29)	−31 (−40; −24)
	74	69 (66–72)	−7 (−11; −3)
	80	86 (80–94)	7 (0; 18)
	157	188 (186–191)	20 (19; 21)

The mass scores are expressed as median and range

The difference between the median and physical mass is also given as median and range

## Discussion

To our knowledge this is the first multivendor study to evaluate the effects of dose reduction and IR on CCS in a dynamic phantom. We have shown that dose reduction in dynamic coronary calcium CT can result in a substantial increase in CCS, whereas the use of IR results in modestly decreased CCS. The most important clinically relevant finding is the ability to reduce dose by 40% in routinely used clinical protocols on state-of-the-art CT systems of four major manufacturers, without compromising the calcium score. This result is not only valid for high plaque burden, but also for the clinically more important mild to moderate coronary plaque burden, represented by the 38 and 74 mg calcifications respectively.

Since risks of radiation dose increase with growing numbers of CT examinations, dose reduction techniques in CCS are highly relevant. Because new guidelines recommend CCS measurements if, after quantitative risk assessment, the risk-based treatment decision is uncertain, it is expected that the number of CT examinations for CCS will further increase in coming years [5]. In the current study we found for all CT systems that dose reductions of 40%, in combination with the in Table 2 specified reconstruction methods, did not significantly affect Agatston scores. For one vendor, the Agatston scores were even similar at 80% reduced dose, and for two vendors there was no significant difference in mass scores at 80% reduced dose in combination with IR.

These results are consistent with those of Hecht et al. [15] who showed in a patient study that for one CT system (equal to S2) CCS can be performed at reduced radiation dose (50%) in combination with IR, without significantly affecting Agatston scores [15]. Ode et al. showed, for a pulsating phantom at 60 bpm and one CT system (similar to S4), that increased IR resulted in decreased Agatston scores, which is in agreement with our results [22]. In comparison with full dose FBP, Agatston scores were not influenced at IR levels L2 and L3 in combination with dose reduction up to 75%, for all used calcifications combined. In our study however, Agatston scores at 40 and 80% reduced dose were found to be significantly different for all IR levels. The reason for this difference is that we only included combinations of dose reduction and IR, when valid for all calcifications separately. Our results also correspond well with a recent study which showed that IR has the potential to reduce radiation dose with 27–54% using a non-dynamic phantom and the same CT systems [23]. With non-dynamic ex vivo human hearts it was shown that a dose reduction of 80% was possible for the four CT systems [23, 24]. This study, however, used static calcifications, did not report on a reference standard of true calcification mass, and used a small-sized phantom. In our dynamic study, we found that a dose reduction of 80% was only feasible for one CT system, and a dose reduction of 40% was possible for all four CT systems, even for low-density calcifications in combination with specific reconstruction methods. Because iterative CT reconstruction significantly reduces calcium scores [10, 25], which potentially alters perceived cardiovascular risk [26], this effect may be counter balanced by the use of reduced dose levels. Moreover, it has been shown that the application of IR significantly improves objective image quality [12], and does not alter quantitative analysis of coronary plaque volume, composition and luminal area [27].

Our results showed a relatively large variation in calcium scores between the CT systems, with Agatston scores ranging from 450 to 738, for the 157 mg calcification. This is in line with previous studies that found that state-of the-art CT scanners of different manufacturers produce substantially different Agatston scores, which can result in reclassification of patients to high- or low-risk categories in up to 6.5% of the cases [28]. Moreover, mass scores generally underestimated the physical mass of the inserts by 3–23% depending on the specific CT system. Underestimations of the physical mass up to 68% were also observed with a static calcium phantom [29].

Reference dose levels, from routinely used clinical protocols of the four high-end CT systems, showed large differences (2.8–10.6 mGy). Despite of these differences in dose levels, similar noise levels were found (22–28 HU). It is important to note, however, that noise is not only determined by dose, but—among other parameters—also by reconstruction kernel. A sharper kernel results in more noise as compared to a softer kernel, if the dose levels are the same. Therefore, different CT acquisition and reconstruction settings may result in different dose levels but similar noise levels. The noise levels behaved as expected as a function of dose reduction and IR: noise levels increased at decreasing dose, and noise levels decreased at increased IR. Our findings indicate that even in the presence of comparable noise levels CCS differed up to 39% between different CT systems at full dose FBP. These differences are surprising for a relatively straightforward metric as the coronary calcium score.

This study has limitations. First, this was an in-vitro study with artificial arteries with calcified inserts. However, the inserts where embedded in an anthropomorphic phantom and were translated at a velocity that is generally observed in in-vivo studies, and the masses of the inserts were in range with calcium masses clinically detected in patients [30]. Second, movement of the calcifications was linear. In vivo, coronary arteries perform a complex movement in three dimensions, which was not feasible in our setup. However, because a linear movement can approximate the movement in 3D during the acquisition time of the CT data, we estimate that addition of 3D movement would result in minor changes in our results. Third, analysis on the inter and intra variability for the different CT systems has not been performed. The associated CT specific correlation between noise reduction and CCS accuracy was also not within the scope of this study. However, these analysis can answer questions about current practice. For example specificity, sensitivity, variations in CCS score between different vendors and the possibility to reduce dose without impact on the metric. Finally, only sequential scan modes were used. With the current appearance of high-pitch spiral mode scanning for coronary calcium it would be interesting to assess the differences in the accuracy of coronary calcium assessment between sequential and high-pitch spiral mode. However, that was not within the scope of this study.

We conclude that for all CT systems a dose reduction of 40% in combination with specific reconstruction gives a CCS comparable for reference protocols. For several systems, even higher dose reductions are possible. Dose reduction results in increased noise and consequently increased CCS, whereas increased IR results in decreased CCS. Mass scores generally underestimated physical mass of the calcifications.

## Figures and Tables

**Fig. 7:**
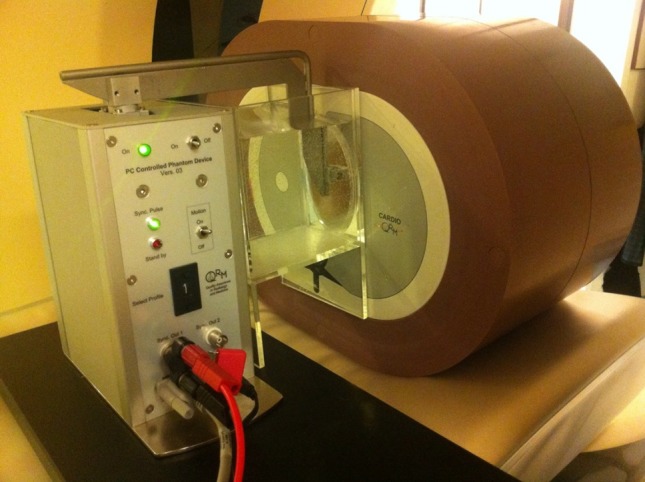
Anthropomorphic chest phantom with extension ring and motion controller (QRM, Moehrendorf, Germany). An artificial coronary artery moved in the horizontal plane inside the water tank in the center of the chest phantom

**Fig. 8:**
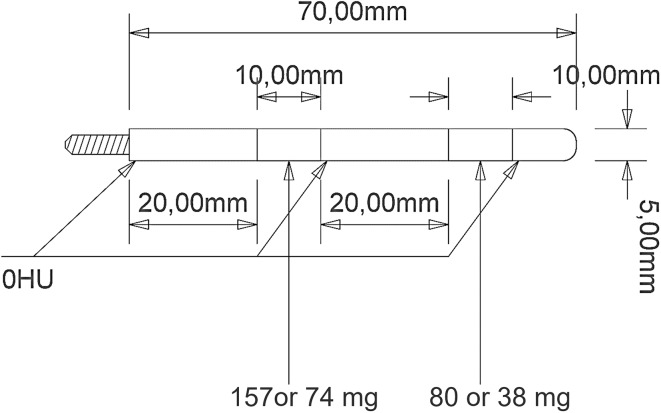
The cylindrical artificial coronary artery contained two calcified inserts with a diameter of 5.0 ± 0.1 mm and a length of 10.0 ± 0.1 mm as indicated

## Appendix 1

See Tables 4 and 5.

**Table 4:** Agatston scores (median and range) for all CT systems, calcification masses, reconstructions and dose values

CT system	Mass	Recon.	Full dose		40% reduction		80% reduction	
			Median (range)	p value	Median (range)	p-value	Median (range)	p-value
S1	38 mg	FBP	95 (92–109)	Ref	109 (99–127)	0.041	150 (137–159)	0.043
		L1	97 (92–107)	0.593	96 (93–108)	0.686	139 (133–147)	0.043
		L2	93 (88–98)	0.042	93 (90–105)	0.141	121 (94–128)	0.225
		L3	91 (87–95)	0.042	91 (88–95)	0.080	94 (88–107)	0.066
	74 mg	FBP	328 (315–345)	Ref	323 (308–336)	0.042	331 (320–345)	0.684
		L1	328 (311–345)	0.102	318 (308–337)	0.068	325 (319–332)	0.498
		L2	326 (306–336)	0.042	305 (296–311)	0.043	314 (303–326)	0.225
		L3	305 (302–323)	0.043	302 (294–307)	0.043	306 (299–311)	0.042
	80 mg	FBP	388 (336–446)	Ref	377 (347–464)	0.893	419 (388–465)	0.043
		L1	388 (333–441)	0.068	376 (329–459)	0.343	409 (381–459)	0.043
		L2	381 (327–441)	0.042	372 (325–452)	0.078	405 (370–446)	0.046
		L3	375 (322–435)	0.043	362 (317–445)	0.043	398 (366–433)	0.225
	157 mg	FBP	497 (490–636)	Ref	595 (561–638)	0.043	664 (602–707)	0.080
		L1	492 (473–631)	0.041	586 (559–623)	0.080	644 (591–692)	0.080
		L2	480 (453–618)	0.043	573 (538–596)	0.138	629 (580–667)	0.080
		L3	463 (432–608)	0.043	569 (528–584)	0.345	616 (566–646)	0.138
S2	38 mg	FBP	102 (90–125)	Ref	106 (89–144)	0.854	265 (202–331)	0.043
		L1	99 (89–107)	0.042	103 (84–112)	0.686	132 (108–209)	0.043
		L2	95 (89–107)	0.043	96 (82–111)	0.104	124 (84–205)	0.225
		L3	95 (89–104)	0.042	95 (69–108)	0.080	99 (70–203)	0.893
	74 mg	FBP	313 (297–342)	Ref	336 (306–351)	0.138	411 (382–514)	0.043
		L1	312 (294–340)	0.041	323 (307–347)	0.225	343 (307–421)	0.043
		L2	295 (284–335)	0.043	317 (297–344)	1.000	315 (294–364)	0.892
		L3	291 (283–329)	0.043	311 (287–337)	0.785	309 (287–354)	0.893
	80 mg	FBP	350 (313–390)	Ref	374 (331–425)	0.225	448 (438–456)	0.043
		L1	349 (307–383)	0.066	376 (328–423)	0.225	389 (371–404)	0.043
		L2	348 (305–369)	0.042	368 (322–412)	0.893	361 (346–373)	0.893
		L3	332 (299–371)	0.068	364 (299–408)	0.893	340 (334–364)	0.500
	157 mg	FBP	505 (439–571)	Ref	551 (449–657)	0.225	690 (472–714)	0.080
		L1	499 (444–568)	0.416	543 (449–652)	0.345	561 (469–652)	0.225
		L2	494 (432–567)	0.043	532 (442–643)	0.345	544 (455–631)	0.345
		L3	490 (433–566)	0.043	520 (434–625)	0.345	528 (446–613)	0.500
S3	38 mg	FBP	102 (80–120)	Ref	105 (93–132)	0.225	151 (145–172)	0.043
		L1	101 (77–118)	0.109	93 (87–116)	0.892	142 (135–161)	0.043
		L2	93 (73–101)	0.043	87 (75–97)	0.080	111 (92–117)	0.225
		L3	80 (72–93)	0.043	77 (70–95)	0.080	88 (85–99)	0.223
	74 mg	FBP	278 (275–309)	Ref	294 (226–311)	0.498	303 (287–377)	0.042
		L1	274 (274–305)	0.042	292 (224–310)	0.715	295 (282–367)	0.042
		L2	273 (267–298)	0.042	267 (202–308)	0.225	281 (248–324)	0.892
		L3	270 (262–278)	0.043	264 (198–302)	0.080	269 (237–312)	0.345
	80 mg	FBP	320 (303–356)	Ref	338 (321–354)	0.138	397 (337–495)	0.043
		L1	318 (298–355)	0.042	332 (313–338)	0.686	391 (334–415)	0.043
		L2	318 (291–330)	0.042	322 (306–331)	0.893	362 (311–378)	0.080
		L3	303 (286–325)	0.043	311 (300–318)	0.225	339 (204–370)	0.686
	157 mg	FBP	450 (420–460)	Ref	459 (425–461)	0.465	561 (517–579)	0.043
		L1	446 (412–456)	0.042	451 (417–453)	0.893	546 (501–552)	0.043
		L2	434 (403–444)	0.043	432 (398–439)	0.345	516 (469–524)	0.043
		L3	414 (398–437)	0.042	420 (392–435)	0.043	484 (450–500)	0.043
S4	38 mg	FBP	109 (93–125)	Ref	107 (94–130)	0.500	186 (166–204)	0.043
		L1	105 (93–113)	0.197	103 (77–107)	0.225	99 (83–112)	0.225
		L2	96 (88–111)	0.080	100 (76–104)	0.068	98 (80–112)	0.225
		L3	88 (82–103)	0.043	97 (75–102)	0.043	82 (75–94)	0.043
	74 mg	FBP	372 (346–391)	Ref	367 (335–397)	0.223	438 (367–462)	0.043
		L1	370 (334–389)	0.336	350 (309–377)	0.043	314 (300–363)	0.043
		L2	365 (327–384)	0.042	350 (308–355)	0.042	308 (297–357)	0.043
		L3	352 (309–367)	0.043	344 (298–350)	0.043	296 (293–314)	0.043
	80 mg	FBP	452 (427–503)	Ref	467 (396–494)	0.225	488 (459–494)	0.892
		L1	462 (423–476)	0.345	445 (392–453)	0.043	402 (370–438)	0.080
		L2	452 (421–480)	0.068	439 (390–451)	0.043	399 (367–438)	0.080
		L3	438 (420–448)	0.042	411 (390–446)	0.043	370 (349–425)	0.043
	157 mg	FBP	738 (672–752)	Ref	706 (603–743)	0.078	716 (659–749)	0.500
		L1	736 (657–744)	0.041	698 (597–726)	0.042	666 (585–675)	0.043
		L2	730 (657–742)	0.066	697 (600–724)	0.043	660 (585–668)	0.042
		L3	736 (660–748)	0.042	694 (606–725)	0.042	654 (584–661)	0.043

P values of the Wilcoxon signed rank test are given for each combination of dose value and reconstruction type, compared to the reference FBP full dose value

**Table 5:** Mass scores (median and range) for all CT systems, calcification masses, reconstructions and dose values

CT system	Mass	Recon.	Full dose		40% reduction		80% reduction	
			Median (range)	p value	Median (range)	p value	Median (range)	p value
S1	38 mg	FBP	23 (20–26)	Ref	25 (20–27)	0.059	26 (21–30)	0.041
		L1	23 (19–26)	0.317	24 (21–26)	0.157	28 (25–29)	0.041
		L2	22 (18–25)	0.034	23 (19–25)	0.043	27 (24–27)	0.038
		L3	22 (16–24)	0.039	22 (16–24)	0.039	26 (21–26)	0.414
	74 mg	FBP	58 (54–62)	Ref	58 (45–62)	0.180	43 (36–54)	0.042
		L1	57 (52–62)	0.059	58 (52–62)	0.180	48 (44–58)	0.039
		L2	57 (55–61)	0.157	58 (52–61)	0.109	53 (50–59)	0.039
		L3	57 (50–60)	0.041	57 (53–60)	0.038	58 (50–58)	0.043
	80 mg	FBP	70 (60–78)	Ref	71 (61–74)	0.892	56 (50–64)	0.043
		L1	69 (61–78)	1.000	70 (65–74)	1.000	58 (51–66)	0.042
		L2	71 (69–76)	0.684	69 (65–74)	0.893	67 (64–72)	0.414
		L3	70 (64–76)	0.680	70 (66–73)	1.000	69 (62–73)	0.785
	157 mg	FBP	125 (108–138)	Ref	110 (105–120)	0.225	111 (108–116)	0.078
		L1	125 (108–138)	0.317	113 (108–118)	0.223	110 (107–116)	0.080
		L2	132 (115–141)	0.077	112 (104–122)	0.225	111 (105–114)	0.080
		L3	135 (118–142)	0.080	119 (112–124)	0.225	112 (111–127)	0.225
S2	38 mg	FBP	25 (22–26)	Ref	24 (22–30)	0.854	43 (34–55)	0.043
		L1	25 (21–26)	0.157	23 (21–28)	0.465	28 (21–38)	0.225
		L2	24 (20–26)	0.180	22 (19–27)	0.257	25 (19–37)	0.500
		L3	23 (20–26)	0.063	22 (18–27)	0.176	24 (17–37)	0.581
	74 mg	FBP	63 (59–68)	Ref	65 (61–70)	0.279	81 (74–101)	0.043
		L1	62 (59–68)	0.157	64 (59–69)	0.892	64 (61–80)	0.684
		L2	61 (58–67)	0.038	63 (58–68)	0.414	60 (58–79)	0.893
		L3	60 (58–66)	0.041	62 (58–67)	0.221	60 (57–77)	1.000
	80 mg	FBP	76 (75–79)	Ref	81 (73–86)	0.138	92 (86–93)	0.043
		L1	75 (75–78)	0.157	80 (72–85)	0.279	76 (71–78)	0.276
		L2	75 (74–78)	0.025	79 (70–84)	0.683	72 (68–74)	0.042
		L3	75 (74–76)	0.109	78 (70–83)	1.000	71 (66–73)	0.042
	157 mg	FBP	165 (161–175)	Ref	174 (163–187)	0.144	200 (181–207)	0.042
		L1	164 (161–174)	0.063	170 (161–186)	0.225	164 (162–188)	0.336
		L2	163 (159–173)	0.025	169 (160–184)	0.498	161 (159–185)	0.498
		L3	163 (159–173)	0.038	168 (159–182)	0.498	160 (157–183)	0.345
S3	38 mg	FBP	20 (16–22)	Ref	20 (18–21)	0.496	24 (23–28)	0.042
		L1	20 (16–21)	0.317	19 (17–20)	0.854	23 (22–25)	0.042
		L2	20 (15–21)	0.083	18 (16–19)	0.197	20 (19–20)	0.684
		L3	19 (14–20)	0.038	17 (15–18)	0.104	17 (16–18)	0.225
	74 mg	FBP	49 (47–53)	Ref	50 (39–54)	0.713	55 (46–60)	0.141
		L1	48 (47–53)	0.317	50 (38–53)	1.000	54 (44–59)	0.225
		L2	47 (47–53)	0.180	49 (38–53)	0.414	50 (42–55)	1.000
		L3	47 (46–53)	0.059	48 (37–52)	0.131	46 (39–52)	0.176
	80 mg	FBP	62 (59–65)	Ref	64 (59–65)	0.357	72 (62–93)	0.068
		L1	61 (59–65)	0.317	63 (59–65)	0.416	71 (61–78)	0.080
		L2	62 (58–65)	0.083	62 (58–64)	1.000	66 (58–70)	0.176
		L3	62 (58–65)	0.083	62 (57–64)	0.892	64 (56–65)	0.713
	157 mg	FBP	131 (128–136)	Ref	132 (129–134)	0.496	145 (138–150)	0.043
		L1	131 (127–136)	0.317	131 (128–133)	1.000	139 (135–146)	0.043
		L2	130 (127–135)	0.025	130 (127–132)	0.680	133 (131–141)	0.273
		L3	130 (127–135)	0.025	130 (126–132)	0.257	130 (126–137)	0.854
S4	38 mg	FBP	26 (23–29)	Ref	27 (23–28)	0.705	32 (29–41)	0.041
		L1	25 (22–28)	0.102	26 (21–27)	0.102	23 (19–27)	0.066
		L2	25 (21–28)	0.102	25 (20–26)	0.068	23 (18–24)	0.068
		L3	23 (20–26)	0.034	24 (20–27)	0.042	21 (17–25)	0.043
	74 mg	FBP	69 (66–72)	Ref	67 (66–69)	0.285	78 (74–80)	0.043
		L1	66 (65–69)	0.066	64 (61–66)	0.042	62 (60–66)	0.042
		L2	65 (64–68)	0.042	64 (61–67)	0.039	60 (59–61)	0.043
		L3	65 (61–66)	0.042	62 (59–65)	0.043	59 (56–62)	0.042
	80 mg	FBP	86 (80–94)	Ref	87 (81–88)	0.496	96 (93–101)	0.039
		L1	85 (79–89)	0.059	82 (76–88)	0.042	79 (76–79)	0.043
		L2	84 (78–87)	0.039	81 (76–85)	0.041	78 (75–80)	0.043
		L3	82 (76–86)	0.034	79 (74–84)	0.042	74 (72–80)	0.042
	157 mg	FBP	188 (186–191)	Ref	185 (176–192)	0.102	197 (189–201)	0.043
		L1	186 (183–194)	0.216	179 (169–186)	0.043	168 (167–173)	0.042
		L2	185 (182–187)	0.039	178 (169–183)	0.043	166 (165–171)	0.042
		L3	181 (178–186)	0.039	175 (168–179)	0.041	162 (160–168)	0.043

P values of the Wilcoxon signed rank test are given for each combination of dose value and reconstruction type, compared to the reference FBP full dose value

## Appendix 2

See Fig. 7.

## Appendix 3

See Fig. 8.

## Abbreviations

CCS: Coronary calcium score
CT: Computed tomography
FBP: Filtered back projection
HA: Hydroxyapatite
HU: Hounsfield units
IR: Iterative reconstruction
